# Optimization of Green Ultrasound-Assisted Extraction of Carotenoids and Tocopherol from Tomato Waste Using NADESs

**DOI:** 10.3390/molecules30030591

**Published:** 2025-01-28

**Authors:** Georgiana Ileana Badea, Florentina Gatea, Simona Carmen Litescu-Filipescu, Andreia Alecu, Ana Chira, Celina Maria Damian, Gabriel Lucian Radu

**Affiliations:** 1 National Institute for Research and Development of Biological Sciences, Centre of Bioanalysis, 296 Splaiul Independentei, P.O. Box 17-16, 060031 Bucharest, Romania; florentina.gatea@incdsb.ro (F.G.); andreia.alecu@incdsb.ro (A.A.); ana.chira@incdsb.ro (A.C.); lucian.radu@incdsb.ro (G.L.R.); 2 Faculty of Chemical Engineering and Biotechnology, National University of Science and Technology Politehnica Bucharest, 1-7 Gh. Polizu Street, 011061 Bucharest, Romania; celina.damian@upb.ro

**Keywords:** lipophilic fraction, carotenoids, tocopherol, NADES, process optimization, UAE extraction

## Abstract

The purpose of this study was to extract the lipophilic fraction from one of the largest source of waste in the industrial sector, namely, the tomato residue from processing the fruit. In order to make this process more environmentally sustainable, this study used a green extraction protocol employing natural deep eutectic solvents (NADESs) combined with a less energy-consuming technology, the ultrasound-assisted extraction (UAE) method, to simultaneously recover carotenoids and tocopherol from dried powder tomato waste. Two NADESs, one hydrophilic and one hydrophobic, were prepared and compared to support high extraction efficiency and increase the stability of the extracted compounds. The optimal extraction parameters were identified as choline chloride:1,3-butanediol (1:5)-based NADES, a solid-to-liquid ratio of 1:20 (*w*/*v*), time of extraction 12 min, temperature 65 °C, radiation frequency 37 Hz, and an ultrasound power level of 70%. The extraction process was intensified and resulted in extracts rich in lycopene (215.13 ± 4.31 μg/g DW), β-carotene (206.95 ± 3.27 μg/g DW), and tocopherol (130.86 ± 8.97 μg/g DW) content, with the highest antioxidant capacity 93.84 ± 0.18 mM Trolox equivalent. Incorporating NADESs for the extraction of bioactive compounds offers numerous benefits, such as improved sustainability, enhanced extraction efficiency, better protection of sensitive compounds, and reduced environmental impact. These advantages make NADESs a promising alternative to traditional organic solvents, especially in industries that require natural, green, and efficient extraction processes for valuable bioactive molecules.

## 1. Introduction

Tomatoes are one of the most popular fruits worldwide, with annual production exceeding 180 million tons. The food industry dedicated to their processing produces large amounts of residue, between 5% and 30% of the initial amount, consisting of the peel, small amounts of pulp, and seeds. This residue is partially used in animal feed, but a vast majority is disposed of on land, creating a major environmental impact. However, tomato residue is a rich source of phytonutrients (carotenes, especially β- and α-carotene, lycopene, lutein, tocopherols, saturated and unsaturated fatty acids, naringenin, and chlorogenic acid) that can be used in the food, medicine, and cosmetics industry [1,2]. The lipophilic fraction of compounds from tomato waste is composed of carotenoids and tocopherols that are highly valuable due to their ability to dissolve in fats and oils, which enhances their bioavailability and antioxidant properties. These compounds offer significant health benefits, including protecting cells from oxidative stress and reducing the risk of chronic diseases, such as heart disease and cancer. In contrast, hydrophilic compounds like vitamins and phenolic acids are water-soluble and have benefits related to immune function and cellular protection. However, lipophilic compounds are often more stable, allowing for better preservation and use in food, cosmetics, and pharmaceutical applications. Carotenoids, such as lycopene, are beneficial for eye health and skin protection, while tocopherols (vitamin E) support immune function and skin health [3]. The use of carotenoids and tocopherols from tomato waste in nanoformulations is an area of growing interest for future studies due to their numerous potential health benefits and sustainable sourcing. By encapsulating these compounds in lipid nanoparticles, they are better protected from environmental factors and can be delivered more effectively to target areas in the body, such as the skin or bloodstream. Studies could investigate the interaction of carotenoids, such as lycopene, and tocopherols (vitamin E) with various lipid materials to determine the most effective formulations that ensure prolonged release, protect the compounds from degradation, and facilitate efficient absorption in the body. Additionally, research could explore the synergistic effects of these antioxidants when delivered together in nanoformulations, potentially offering enhanced therapeutic outcomes, such as improved skin protection, anti-inflammatory effects, and prevention of chronic diseases.

Finding methods to valorize these compounds through non-polluting, selective processes with low energy consumption is a priority in the context of both innovation and the recirculating economy [4].

Therefore, in parallel with the promotion of new types of green solvents (e.g., liquid ionic solvents (ISs), deep eutectic solvents (DESs), natural deep eutectic solvents (NADESs), biobased solvents represented by ethanol, glycerol, and terpenes), environmentally friendly extraction techniques have also been developed, the most promising being considered ultrasound-assisted extraction (UAE), supercritical fluid extraction (SFE), microwave-assisted extraction (MAE), and accelerated solvent extraction (ASE). While ISs have already been regulated in certain countries due to their toxicity, the newest practical applications in the food, cosmetics, or pharmaceutical industry are related to NADESs derived from DESs, which represent one of the most promising categories of natural solvents due to their special properties: high biodegradability, low volatility, high polarity, moderate-to-low toxicity, and low cost of production [5]. NADESs are considered “natural” because the components in their structure are part of primary metabolites (such as sugars, organic acids and bases, and amino acids) that are used by the plants themselves for survival or can be easily found on the profile market. In plants, NADESs act as solvents to dissolve metabolites, enzymes, and membranes, contributing to the stability of the cellular environment during extreme temperature changes. Their importance is also enhanced by the diversity of chemical compounds that can be extracted in NADESs, and thus both hydrophilic and hydrophobic compounds can be extract separately or at the same time. Eutectic mixtures with organic acid components have the highest polarity, followed by those based on amino acids. From a chemical point of view, the increased solubility of some compounds in NADESs is the result of dipole–dipole interactions and hydrogen bonds that form in the NADES–solute interaction [6]. However, there is also a major disadvantage of NADESs compared to conventional solvents, namely, their high viscosity, which can reduce the diffusion coefficient of the analytes, affecting the extraction yield and leading to increased time of extraction. This disadvantage can be minimized by the possibility of adding water to some NADESs and by increasing the extraction temperature. On the other hand, NADESs can play an important role in small-molecule formulations (e.g., hydrogels) or as a vehicle of liposoluble bioactive compounds [7].

The efficiency of NADESs has been demonstrated both in the extraction procedures of various plant materials and for the valorization of bioactive compounds from industry waste. A NADES consisting of lactic acid and glucose (5:1) with 20% water content proved to be a more effective extraction medium for bioactive compounds from mango peel waste. At an extraction ratio of 30:1 (*v*/*w*), UAE extraction for 30 min, the NADES resulted in a 1.4-fold higher total polyphenol content, a 1.7-fold higher flavonoid content, and an antioxidant activity 1.9 times higher than in the case of classic extraction with 80% ethanol [8]. In the case of grape skin and seed waste, a NADES consisting of choline chloride, levulinic acid, and ethylene glycol (1:1:2) with 20% water led to an increase in the extraction yield of ellagic acid by 1.73 times in comparison with the classic extraction in 75% ethanol, and the combination of choline chloride, proline, and malic acid (1:1:1) with 30% water allowed the extraction of the largest amounts of catechin and epicatechin [9]. The efficiency of NADESs in the extraction of anthocyanins from various raw materials has also been reported, these solvents leading to high extraction yields [10,11].

Other types of food waste that offer a source of bioactive compounds are those resulting from the processing of tomatoes and various vegetable materials that contain carotenes (pumpkin, carrots, etc.). A combination between two fatty acids C8 and C10 (1:2) allowed the extraction of lycopene and β-carotene in a quantity comparable to the extraction in acetone for tomato peel waste [12,13]. The same combination in a different ratio (3:1) was used for the extraction with a high yield of β-carotene from pumpkin [14]. By using a NADES (lactic acid–glucose, 5:1) on tomato peel waste, an extract with high rutin, caffeic acid, quercetin, naringenin, and catechin content was obtained [15].

Since the most used techniques for lipophilic compound recovery from waste and by-products are being carried out with solvents from the petrochemical industry (which are highly flammable, corrosive, carcinogenic, and harmful substances), the use of green solvents and combinations with other extraction procedures may lead to a safe extract that can be integrated into food products or cosmetics without elimination of residual solvents.

Therefore, the aim of this study was to optimize a simple and safe method for simultaneous recovery of the lipophilic fraction from tomato residue combining UAE and NADESs. The main goal of the extraction method was to obtain a balanced compromise between carotenoid content, tocopherol content, and antioxidant capacity, because these three factors contribute significantly to the nutritional and functional properties of the extracts that could be further used as natural food pigments or/and nutraceutical ingredients. An HPLC-PDA method was employed to characterize and quantify the lipophilic profile and the carotenoids (lycopene, β-carotene, and lutein) and tocopherol content in the tomato waste extracts. The antioxidant activity of the extracts was evaluated using the spectrophotometric method with 2,2′-azino-bis (3-ethylbenzothiazoline-6-sulfonic acid) (ABTS).

## 2. Results and Discussion

### 2.1. Characterization of NADESs

The density of NADESs is an important parameter that has a great impact on their physicochemical properties and can affect the extraction efficiency through implication in mass transfer and heat transfer processes [16]. Table 1 shows the density of NADESs, which are 1.0465 g/cm^3^ for DES-1 and 0.9061 g/cm^3^ for DES-2. It is well known from the literature that most of the hydrophobic DESs have density lower than water, in our case 0.9061 g/cm^3^ for a menthol-based DES [17].

**Figure 1:**
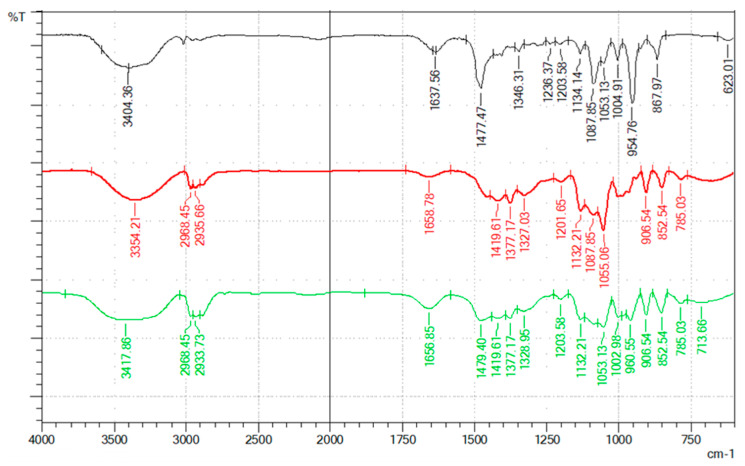
FTIR spectra of pure choline chloride (black spectrum), 1,3-butanediol (red spectrum), and the prepared NADES-1 (green spectrum).

**Figure 2:**
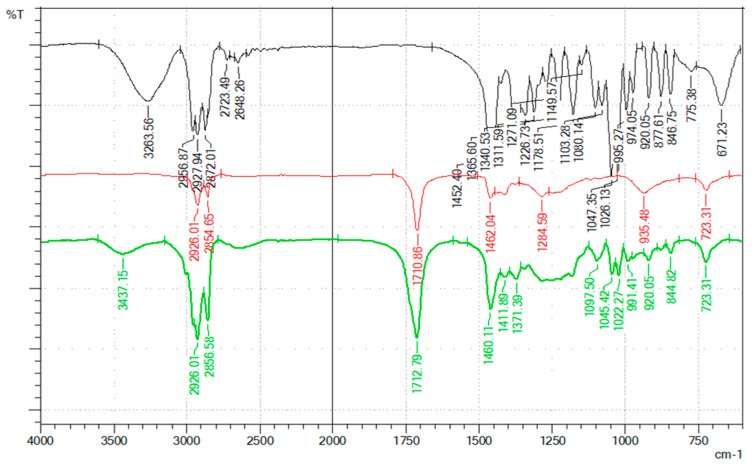
FTIR spectra of pure menthol (black spectrum), oleic acid (red spectrum), and the prepared NADES-2 (green spectrum).

In order to understand the hydrogen bond interactions between HBA and HBD, an FTIR analysis was conducted, and the results are shown in Figure 1 and Figure 2 as spectra of the two NADESs (DES-1 and DES-2) and their pure components (choline chloride, menthol, 1,3-butanediol, and oleic acid). Figure 1 presents the overlayed FTIR spectra of ChCl (black spectrum), 1,3-butanediol (red spectrum), and NADES-1 (green spectrum).

The investigated ChCl spectrum revealed that the fingerprint region had two prominent peaks at 1087 and 954 cm^−1^, identified as the stretching of C-N of the quaternary ammonium compound and decreasing in intensity during NADES formation. The choline chloride did not have any active vibrational mode in the 1700–1600 cm^−1^ region, and therefore the band observed at 1637 cm^−1^ is solely attributed to the water scissor mode. One broad band at 3404 cm^−1^ that belongs to O-H stretching vibration and a vibration band around 3000 cm^−1^ that can be attributed to the stretching mode of C-H stretching vibration are shown in the ChCl spectrum. The band in the 3500 cm^−1^ regions from choline chloride and 1,3-butanediol spectra broadens in the NADES spectrum at 3417 cm^−1^, the shift indicating the formation of hydrogen bonds between the two components. The bands from 2968 cm^−1^ and 2933 cm^−1^ indicate the presence of C-H stretching. The sharp band at 1477 cm^−1^ refers to the alkyl group that is present in choline chloride and is reconfirmed in the NADES spectrum at 1479 cm^−1^, confirming the characteristic of choline chloride [18].

The changes in bond length and corresponding vibrations of hydrogen bonds can indicate the formation of hydrogen bonds [19]. It can be seen that the spectrum of NADES-1 presented in Figure 1 was roughly an overlap of the choline chloride and 1,3-butandiol, especially the vibration band that corresponded to the amino group (800–1200 cm^−1^).

The examination of the FTIR spectra for the prepared NADES-2 in Figure 2 confirmed the intermolecular hydrogen bond interactions between menthol and oleic acid.

The FTIR spectrum of menthol crystals (black spectrum) shows strong to medium absorption bands at 3264 cm^−1^ assigned to –OH stretching vibrations, 3050–2750 cm^−1^ (C–H symmetric and asymmetric stretching vibrations), 1452 cm^−1^ (–OH deformation vibrations and –CH2 scissoring vibrations), 1400–1200 cm^−1^ (C–H wagging vibrations), and 1100–1000 cm^−1^ (C–O stretching vibrations) due to functional groups and lattice vibrations [20,21]. For NADES-2, the –OH absorption band is broader and is shifted at higher frequency (3437 cm^−1^) due to involvement in strong hydrogen bonds [20]. The intensity of absorption bands assigned to C–H symmetric stretching vibrations decrease is due to a reorganization in a less crystalline structure. In the 1500–1150 cm^−1^ region, there is an overlap of the absorption bands due to the O–H and C–H vibrations, this making the spectrum difficult to interpret. The absorption bands from 1100–1000 cm^−1^ region are changing their form and frequency, this being proof of the involvement of functional groups in the hydrogen bond formation. For oleic acid, the FTIR spectrum suffers changes in the main absorption bands assigned to C=O stretching vibrations (1713 cm^−1^) and O–H···O out of plane deformation (935 cm^−1^) from the carboxylic group involved in hydrogen bons. The conjugation has small effects on the stretching vibration, and the absorption band becomes broader [21]. Similar changes in these regions, and especially shifts of the O–H and C=O bonds to higher wavelengths, have been reported in the literature (e.g., [19]) and can be considered confirmation of the formation of a hydrogen bond network between the individual components to form NADESs.

**Figure 3:**
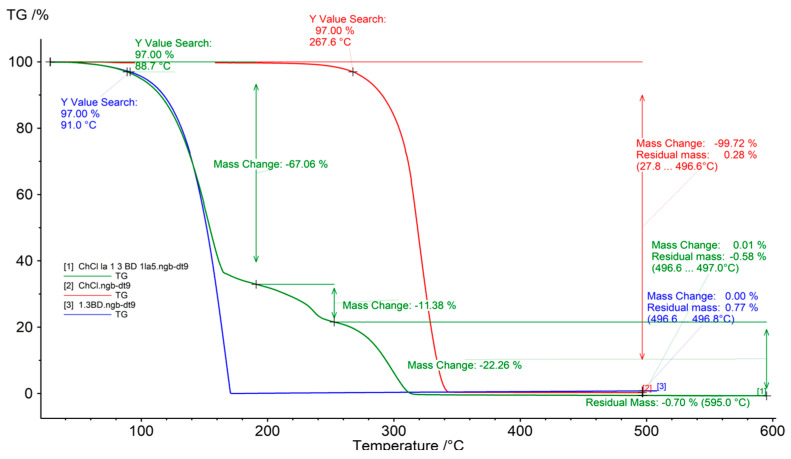
Thermogram of NADES 1,3-BD–ChCl (1:5). The x-axis shows the increase in temperature (°C), while the y-axis shows the loss in weight (%).

The evaluation of eutectic solvent thermal stability is important, since it provides information about the temperature at which the solvent can be used without suffering alterations. Figure 3 presents a thermogram measured to determine the degradation temperatures of the hydrophilic NADES-1 and its components, and represents the weight loss as a function of temperature. As can be seen in Figure 3, the hydrophilic NADES-1 presented a two-step decay of weight loss, about 67% below 180 °C, which was mainly due to the degradation of 1,3-butanediol. The NADES sample gradually started weight loss at 170 °C and completely decomposed at about 310 °C. Therefore, NADES-1 remained thermally stable in the studied temperature range of 50–100 °C. For this sample, the second plateau is probably caused by different degradation temperatures of the two components that form the mixture. For NADES-2, the sample shows a weight loss below 200 °C, with a complete degradation of its components at 120° for menthol and 220° C for oleic acid.

### 2.2. Selection of the Most Efficient NADES for Lipophilic Compound Recovery from Tomato Waste

**Figure 4:**
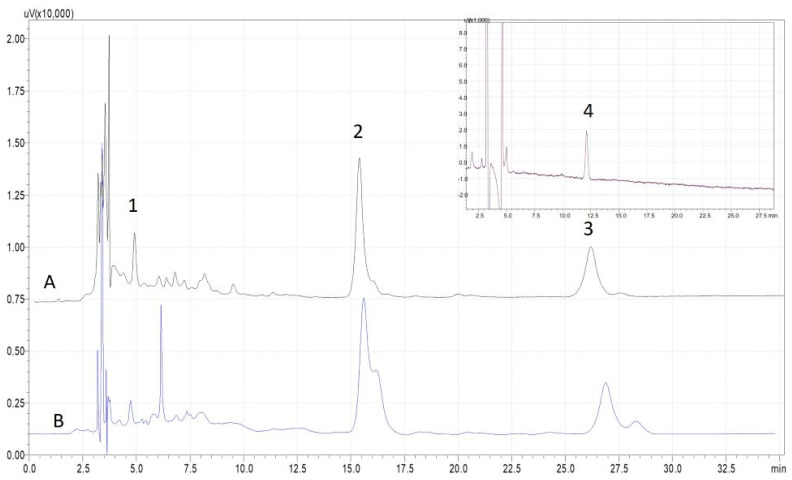
HPLC profile of the major carotenoids (1-lutein, 2-lycopene, 3-β-carotene, at 458 nm) and tocopherol (4-inset, at 293 nm) in tomato waste extracts using NADES-1 (**A**) and NADES-2 (**B**) as extraction solvents: 30 min time of extraction, temperature 55 °C, ultrasound power 100 W, radiation frequency 37 Hz, and S/L 1:15 (*w*/*v*).

The resulting extracts were characterized using HPLC-DAD, and representative profiles are presented in Figure 4. The results along with the antioxidant capacity assessment are presented in Table 1. The two solvents exhibited similar profiles, with the peaks attributed to lycopene and β-carotene being significantly more abundant than others. There are three distinct chromatographic peaks ascribed to lutein, β-carotene, and lycopene, but using the hydrophilic NADES, another liposoluble compound was extracted, namely, tocopherol. This compound was not found in the extract obtained using the menthol–oleic acid composition. The concentration of β-carotene and lutein in the NADES-2 extract (273.60 and 4.12 μg/g DW respectively) was double that of the NADES-1 extract (141.64 and 2.16 μg/g DW respectively). Even though lycopene is the main carotene in tomato fruit, it also contains a significant amount of β-carotene, formed by the action of the enzyme lycopene beta-cyclase, which transforms lycopene through a cyclization mechanism, and this could be an explanation of the higher content of β-carotene in the NADES-2 extract [22,23].

Chromatograms representing the carotenoid profile in the tomato waste extracts are shown in Figure 4.

Although the best results in terms of total content of bioactive compounds were obtained with solvent NADES-2 (476.57 μg/g DW), the presence of the tocopherol compound (117.74 μg/g DW) and the highest antioxidant capacity (65.11 mM Trolox eq.) in the first extract fulfilled the most important criterion of the study, namely, obtaining a balance between extraction efficiency, yield, and compound stability. As a consequence, the study proceeded further with optimization of the UAE parameters using the NADES-1 solvent, namely, chlorine chloride–1,3 butanediol.

**Figure 5:**
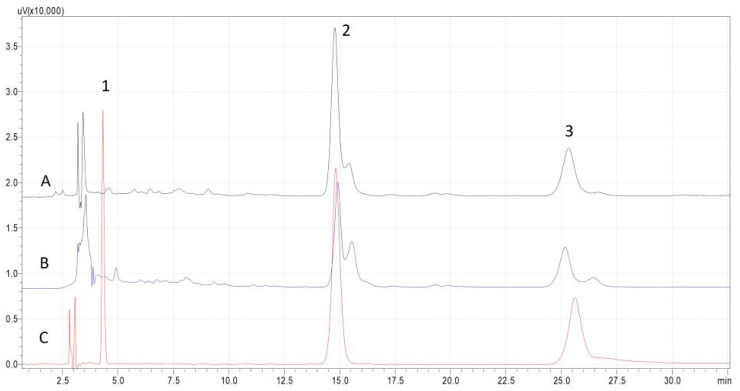
Comparison of HPLC chromatograms for different extraction solvents—sunflower oil (**A**) andNADES-2 (**B**)—with the chromatogram of a mixture of standards (**C**) at 458 nm (1-Lutein, 2-Lycopene, 3-β-carotene).

As can be seen in Figure 5, an explanation is required based on the chromatograms of the extracts obtained by UAE using the green solvents, NADES-2, and sunflower oil for liposoluble compound recovery.

When comparing the chromatogram of a mixture of standards with the chromatograms of the extracts, a slight difference can be seen, such as additional peaks or small shifts in retention times for the carotenoids of interest. Standard solutions typically contain only specific isomers, whereas extract samples may have a mixture of them, resulting in a more complex chromatographic profile. In Figure 5, a weak shoulder appears at the chromatographic peaks assigned to lycopene and β-carotene. Carotenoids from UAE extracts may undergo degradation or modification due to factors that are optimized during extraction, like high temperature, long exposure to ultrasounds, light exposure, or oxidation reactions. These modifications appear in the chromatograms as tailored peaks, double peaks, or shoulders to the main peak. Also, the carotenoid class of compounds exist in cis/trans isomeric forms in nature, and therefore the ratio of these isomers can vary in real samples compared to the standard solution. Due to slightly different physicochemical properties (e.g., polarity) of these isomer forms, they can elute at different times during chromatographic separation, making the interpretation a little difficult. It is known from literature data that heat can induce isomerization of the all-trans to cis lycopene forms [24]. This may explain the appearance of peaks alongside the pure standard peak of lycopene (15-cis- and 9-cis-isomers of lycopene are more abundant in heated rather than in raw vegetable material). In this study, there was no possibility of using pure standards of other isomers of the targeted carotenoids: they were quantified using the calibration curves of the available compounds, and the content was combined with total carotenoid content.

### 2.3. Optimization of the UAE Parameters for Lipophilic Compound Recovery from Tomato Waste

Carotenoids, such as beta-carotene and lycopene, are sensitive to environmental factors like heat, light, and oxygen. Extraction parameters like temperature, solvent type, and time can influence carotenoid retention or degradation. If the conditions are extreme (e.g., high temperatures or prolonged time of extractions), carotenoids can degrade, reducing in content. The tocopherols, including vitamin E, are also sensitive to heat and oxidative degradation. Extracting them requires careful control of temperature and time to preserve their concentration. Over-extraction or improper solvent selection can lead to the loss of tocopherols, reducing the antioxidant efficacy of the extract. Both carotenoids and tocopherols contribute to the overall antioxidant capacity of the plant extract. Antioxidant capacity is crucial for health benefits, such as neutralizing free radicals and preventing cellular damage. The extraction process must therefore be optimized not just to extract high levels of carotenoids and tocopherols but also to preserve their combined antioxidant potential. The most appropriate time and temperature of extraction along with the compatible solvent ensures that these compounds are efficiently extracted while maintaining their synergistic antioxidant effects. This results in higher-quality extracts with better health properties, which is essential for applications in food, cosmetics, and pharmaceuticals.

Therefore, starting with Table 2, the results obtained while optimizing the HBA–HBD ratio with respect to individual and total content of lipophilic compounds extracted are presented. The best NADES component combination, reaching extraction efficiencies higher than 300 μg/g DW extract, can be rated according to the following order for molar ratio: 1:4 > 1:5 > 2:3 > 1:2 > 1:3. The best solvent combination for extracting lycopene was the molar ratio 2:3 (ChCl–1,3-butanediol) when 146.63 ± 8.23 μg/g DW of lycopene was recovered, while for the β-carotene, lutein, and tocopherol recovery, the strongest solvent was chlorine chloride–1,3-butanediol at a molar ratio of 1:5. Although the results are contradictory when it comes to obtaining the highest lycopene or β-carotene content, the best antioxidant capacity was obtained when a HBA–HBD ratio of 1:5 was used. Taking into account the compromise regarding the total content of liposoluble compounds, implicitly possessing the best antioxidant activity, the study moved forward with optimizing the extraction parameters using a solvent whose components were used in the respective 1:5 molar ratio combination.

**Figure 6:**
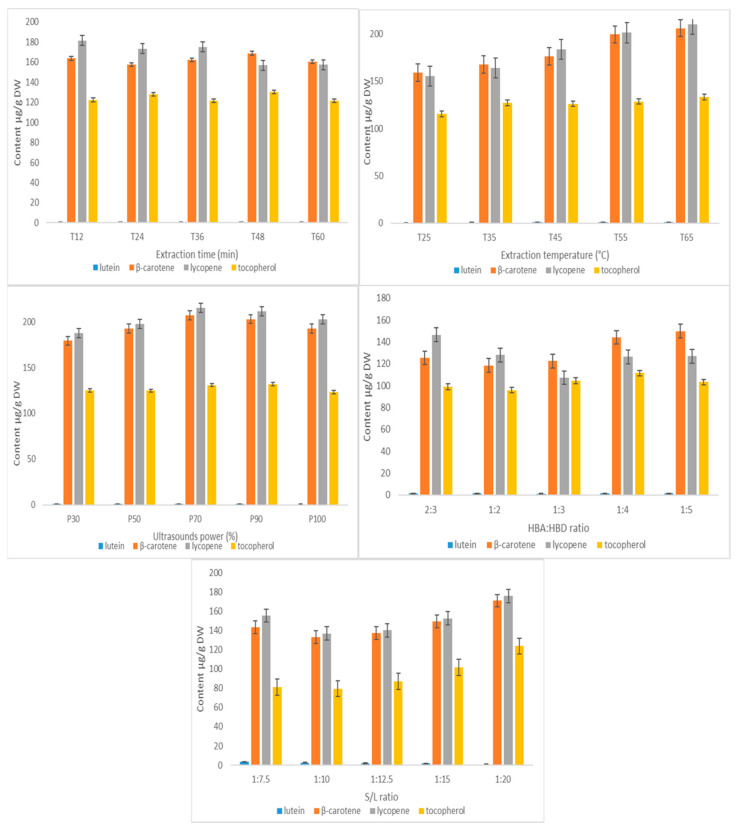
Optimization of carotenoid extraction from tomato waste using different parameters (NADES component molar ratio, solid-to-solvent ratio, time of extraction, extraction temperature, and sonication power). The extraction conditions are given in Table 2, Table 3, Table 4, Table 5 and Table 6.

An optimum ratio of S/L is required to allow proper mass transfer, and consequently produce excellent extraction yields. This key parameter significantly impacts the overall cost of the extraction technology and the waste management therefore, the effect of solid-to- liquid ratio on the extraction efficiency of lipophilic compounds was experimented while maintaining the other extraction parameters constant (time of extraction 30 min, temperature 55 °C ultrasound power level 100%, radiation frequency 37 Hz, HBA:ABD ratio (1:5)). Table 3 presents the results and along with graphical representation in Figure 6.

When the S/L ratio decreases, the total lipophilic compound content decreased until the 1:12.5 ratio but further, the bioactive compound extraction efficiency increased, obtaining the highest carotenoid content around 342.13 μg/g DW using the 1:20 (g/mL) S/L ratio. This phenomenon may be attributed to the increased contact area between the solute and the solvent, which reduced the density of the mixture and increased the speed of ultrasonic propagation, thereby improving the dissolution rate. However, there was an observation in this step regarding the lutein and tocopherol extraction efficiencies, namely, that the content for this xanthophyll decreased with decreasing the S/L ratio, while for tocopherol the amount increased with decreasing S/L ratio. Therefore, the liquid–solid ratio of 20 mL/g was selected for the next steps of the experiment.

This study investigated the yields of total lipophilic fraction recovered from tomato waste when exposed between 12 and 60 min of irradiation while keeping other parameters constant (time of extraction 30 min, temperature 55 °C, ultrasound power level 100%, radiation frequency 37 Hz, HBA–ABD ratio 1:5, S/L 1:20 (*w*/*v*)). As shown in Figure 6, the extraction yield decreased with time and reached its maximum at 12 min exposure, with a value of 468.03 ± 17.01 μg/g DW for total content of bioactive compounds.

As can be seen from the results (Table 4), the extraction yield decreased as the ultrasonication time continued to increase. When it comes to individual compound content, there are a few remarks to be made, namely, that the β-carotene content did not vary much by the time of extraction, ranging from 157.68 ± 7.10 to 168.78 ± 2.20 when the time doubled. The tocopherol content was also very stable when the time doubled, e.g., for extraction at 12, 36, and 60 min, the amount of tocopherol recovered stayed around 121–122 μg/g DW, with a slight increased for 24 and 48 min, when it reached 130.02 ± 3.65 μg/g DW. Lycopene itself is the only compound that loses approximately 14% of its content by increasing the time of extraction, which is in agreement with other studies on carotenoid stability when subjected to thermal processes [24].

Generally, a later extraction can enhance the adequate dissolution of bioactive compounds in the solvents. However, extended exposure to ultrasound may lead to structural degradation of carotenoids in the extracts due to overheating of the media. It is well known that carotenoids are susceptible to degradation in the presence of heat, light, and oxygen. Hence, prolonged ultrasonication before extraction is not recommended for labile carotenoids. These results also confirm that ultrasound has the ability to dissolve compounds from plant cell walls within a relatively short period. Similar behavior was observed in a study on ultrasound-assisted natural deep eutectic solvent extraction of lycopene from pumpkin [25]. Therefore, the time of extraction of 12 minutes using ultrasonication was chosen for further optimization of the process.

The last parameter for optimizing the extraction process is the appropriate extraction temperature, which is critical for the efficient recovery of heat-sensitive liposoluble compounds due to its impact on the structural integrity, as well as on the viscosity of the NADES [26]. In this study, the effect of ultrasonication temperature on the extraction efficiency was tested from 25 °C to 65 °C while keeping other parameters constant. The results are shown in Figure 6 and Table 5.

The ultrasonication temperature had an obvious effect on carotenoids yields: as the temperature increased, the extraction yield of carotenoids showed an increase and reached its maximum at 65 °C. A possible explanation for this phenomenon is that by increasing the temperature, the viscosity of the NADES may decrease, leading to an increase in the mass transfer rate. Since in the process of ultrasound-assisted extraction, both the thermal and the cavitation effect play a significant role, the cavitation effect may be enhanced by increasing the temperature, thereby improving the extraction efficiency. However, as the temperature continues to rise, the carotenoids might suffer degradation processes when exposed to excessive temperatures, as they are known as thermosensitive compounds. Therefore, an extraction temperature of 65 °C was selected for the follow-up experiment.

The last parameter that was investigated for the influence on the extraction process was the sonication power. Ultrasonic techniques are classified into different groups depending on the ranges of frequency and power during operation. Combination of high frequency and low power is characteristic for diagnosis in medicine, whereas the combination of low frequency and high power is classified as power ultrasound and is known to produce cavitation effects in extraction materials or mixtures and enhance the separation of particulates from biological matrices [27]. The experimental data are shown in Table 6 and present the carotenoid content obtained at different ultrasonic power levels (30% to 100%). As can be seen from the results, the highest extraction efficiency for total carotenoid content was obtained using 70% ultrasonic power (equal to 210 W) with 553.84 ± 16.61 μg/g DW lipophilic compounds in the extract. With respect to this parameter, we observed that by increasing the power level, the individual and total content of the targeted analytes increased exponentially. The carotenoid yield of tomato extracts increased by 11.4% when power was increased from 90 to 210 W. Changes in the chemical composition of extracts were observed when the power increased towards 100% (300 W), while total carotenoid content decreased by 6%, reaching 518.75 ± 16.96 μg/g DW at the highest power.

Figure 6 summarizes the experimental data obtained during the optimization of the extraction process.

The ANOVA results applied to all the experimental data show a highly significant effect between the variables (Table 7), with an F value of 647.06 and a *p*-value of 5.64 × 10^−52^, proving substantial differences between the amount of individual compound and the different conditions of extraction.

Since the *p*-value corresponding to the F-statistic of the ANOVA was lower than 0.01 and strongly suggested that one or more pairs of treatments (experimental conditions) were significantly different, Tukey’s HSD test was applied to pinpoint which of them exhibited statistically significant difference. The results indicated that the only differences for all the compounds between the experimental conditions were the HBA–HBD ratio versus time of extraction, solid-to-liquid ratio versus the temperature of extraction, and solid-to-liquid ratio versus the ultrasound power levels.

Table 8 present the results obtained using UAE and ASE with different conventional and green solvents (sunflower oil, ethanol, organic solvent mixture: hexane–acetone) in order to compare the extraction efficiency of the investigated compounds. The extraction conditions were those optimized in the previous section, and the results are expressed in µg/g DW. Our results were comparable with those obtained when using the characteristically organic solvents for liposoluble compounds and the ASE method in terms of total content of antioxidant compounds and higher extraction yields when compared with a greener solvent, ethanol 96%. As a conclusion, a lower extraction yield was obtained, especially in the case of ASE, which is most likely due to the short time of extraction (three cycles/5 static minutes) and the low temperature at extraction (the extractions took place at ambient temperature). This is in agreement with experiments carried out to optimize the extraction parameters: a higher temperature (approximately 60–65° C) ensured a higher extraction efficiency, especially of the compounds from the liposoluble class. When using sunflower vegetable oil and UAE, significant amounts of carotenoids are obtained, almost double quantities for lycopene and β-carotene, and approximately 20 times more tocopherol. Regarding this high content of antioxidant compounds in sunflower oil extracts, there is plenty of information in the literature on the contribution that the solvent (in this case, sunflower oil) also brings in terms of the content of fat-soluble vitamins in the extracts formulated with these green solvents [28].

Table 9 presents comparative quantitative results reported in the literature for the extraction of lycopene and carotenes from tomato residue using various methods. As can be seen, the bioactive compounds amount obtained by UAE–NADES-based extraction developed in this research study are comparable to those obtained by other modern and conventional methods.

Incorporating NADESs for the extraction of bioactive compounds offers numerous benefits, such as improved sustainability, enhanced extraction efficiency, better protection of sensitive compounds, and reduced environmental impact. These advantages make NADES a promising alternative to traditional organic solvents, especially in industries that require natural, green, and efficient extraction processes for valuable bioactive molecules.

## 3. Materials and Methods

### 3.1. Vegetable Material

**Figure 7:**
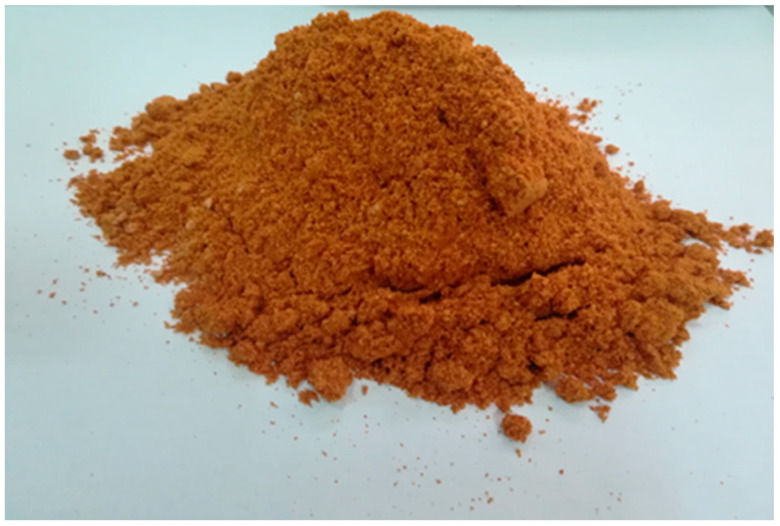
Dried tomato powder used for UAE extraction with NADESs.

The tomatoes were purchased from the local market. Separation of the seeds and peels was accomplished with the help of a household device used in the preparation of tomato juice. The residue was dried in an oven (Pol-Eko Aparatura, Wodzisław Śląski, Poland) at 37 °C and then crushed in a knife mill (Grindomix GM100, Retsch, Haan, Germany) to a particle size of <1 mm. The ground peels and seeds (powder form, Figure 7) were stored in Ziploc bags wrapped in aluminum foil at −20 °C until analysis.

### 3.2. Chemicals and Reagents

All solvents used for the extraction and column chromatography were of analytical grade (maximum required purity). Lycopene (≥98%, HPLC), (±)-α-tocopherol (≥96%, HPLC), oleic acid, and β-carotene CRS were purchased from Sigma-Aldrich (St. Louis, MO, USA). Lutein was obtained from PhytoLab (Vestenbergsgreuth, Germany). DL-menthol and choline chloride (≥98%) were purchased from TCI (Tokyo Chemical Industry Co., Zwijndrecht, Belgium), and 1,3-butanediol was purchased from ROTH (Carl Roth Gmbh, Karlsruhe, Germany). The organic solvents—methanol, ethanol, acetone, n-hexane, acetonitrile, and dichloromethane—were HPLC grade and obtained from LiChrosolv (Merck, Darmstadt, Germany). Other reagents used in this process were butylated hydroxytoluene (BHT, 99.0%) and triethylamine (TEA), also purchased from Sigma-Aldrich GmbH (Steinheim, Germany).

### 3.3. Screening of NADESs for the Extraction of Lipophilic Compound Fraction of Tomato Waste

Preliminary experiments were conducted to select the optimal NADES for extracting the lipophilic compound fraction from tomato waste. The components evaluated to prepare the NADESs were choline chloride combined with 1,3-butanediol (ChCl-But) and DL-menthol combined with oleic acid (Men-OA) in order to compare a hydrophilic solvent with a hydrophobic one with respect to the extraction yields. The extraction was performed at the constant parameters presented in Section 3.6. The extracts were centrifuged for 15 min at 5000 rpm, and the supernatant was collected and diluted with acetone–methanol (75:25, *v*/*v*) at a 1:1 ratio for further quantitative analysis. All manipulations were carried out in dim light to minimize photodegradation of carotenoids throughout the analytical procedure. Two successive extractions under the same experimental conditions using the two NADESs chosen were also carried out for reproducibility evaluation. The investigated NADESs were compared in terms of individual compound content and antioxidant capacity of the resulting extract using chromatographic and spectrophotometric methods. All experiments were performed in triplicate, and the results are expressed as means ± standard deviation.

### 3.4. Preparation of NADESs

**Figure 8:**
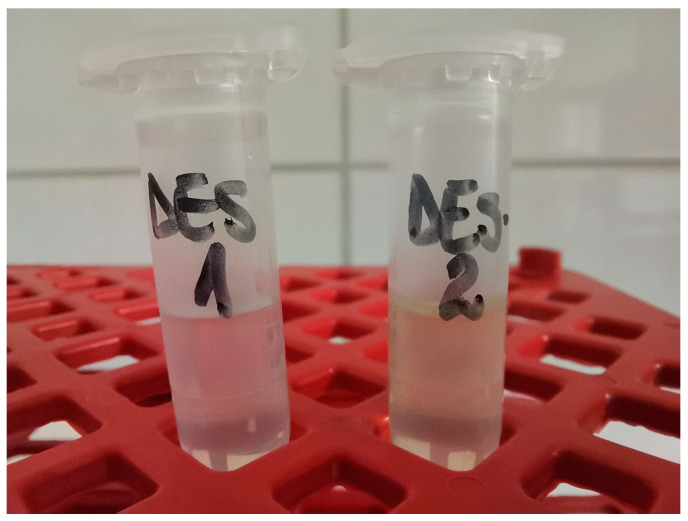
The investigated mixtures at room temperature (compositions defined in Table 10, NADES-1 left, NADES-2 right).

NADESs were prepared by mixing appropriate ratios of hydrogen bond acceptors and hydrogen bond donors (see Table 10, Figure 8). The mixtures were heated to between 50 °C and 100 °C using a magnetic stirring–heating plate at 900 rpm for 1–4 h until a transparent homogeneous liquid was obtained. In the case of menthol-based NADESs, a yellowish transparent homogeneous liquid was obtained. After cooling slowly to room temperature, the NADESs were used either within the same day or stored at room temperature for further use.

### 3.5. Characterization of NADESs

The prepared solvents were characterized in terms of density and by Fourier transform-infrared spectroscopy (FTIR) in order to demonstrate the formation of hydrogen bonding interactions between the initial components. Density tests were performed following a simple gravimetric procedure using a calibrated volume at 23 °C.

IR spectra were acquired using an IRTracer 100 Shimadzu spectrophotometer (Shimadzu Europa GmbH, Duisburg, Germany) equipped with an RT-DLaTGS detector and KBr beam splitter. The spectral range was set between 4000 and 600 cm^−1^ in the transmittance mode, and for each spectrum, a total of 45 scans with a spectral resolution of 4 cm^−1^ were acquired at a scanning speed of 2.8 spectra/second. The original spectra were corrected for CO_2_ and H_2_O and subjected to smoothing and baseline correction with the aid of LabSolution software (version 1.25).

The thermal behavior of the NADESs was studied using thermogravimetric analysis (TGA) and differential scanning calorimetry (DSC). Thermogravimetric analysis (TGA) was performed using a Netzsch TG 209 F1 Libra (Netzsch-Gerätebau GmbH, Selb, Germany) equipment, from RT to 800 °C under nitrogen atmosphere with a heating rate of 10 °C/min.

### 3.6. Optimization of Extraction Parameters for the Lipophilic Fraction of Tomato Waste Using UAE and NADESs

Besides the use of green solvents, one of the criteria for green extraction is to reduce energy consumption by using innovative technologies such as UAE and ASE [35]. In order to optimize the extraction method, after selecting the appropriate NADES, the following extraction parameters were varied: the power of ultrasound, solid–liquid ratios (*w*/*v*), extraction temperature, and time of extraction. The extraction temperature is an important factor affecting the UAE efficiency. An increase in temperature causes a decrease in the viscosity and diffusivity of the solvent, which is very important for viscous solvents such as DES. In general, at higher temperatures, the solvent power increases and a reduction in surface tension occurs, leading to a stronger interaction between target compounds and sample matrix and improving dissolution in the specific solvent [36]. On the other hand, it is well known that high temperatures cause thermal degradation of carotenoids compounds, and therefore we decided to study a moderate temperature range (25–65 °C). In addition to the abovementioned factors, the time and the irradiation power also play an important role, because the effect of ultrasound cavitation may accelerate or trigger chemical reactions in the extraction medium, leading to unwanted results. Therefore, the irradiation power was varied between 30 and 100 W and the time ranged from 12 to 60 min.

The solid-to-solvent ratio is another critical parameter in demonstrating the environmental sustainability of the extraction methods, since it influences the energy consumption and the production of waste at the end of the technological process. In this study, the S/L ratio was varied from 20:1 to 7.5:1, with the other parameters constant.

The extractions were performed with an ultrasonic bath Elmasonic P (Elma Schmidbauer GmbH, Singen, Germany) that offers the possibility of selecting the ultrasound power, frequency, and temperature in the bath. At first, the extraction was carried out for 30 minutes at a temperature of 55 °C and an ultrasound power level of 100%, while the radiation was at a fixed frequency of 37 Hz for a solid-to-solvent ratio of 1:15 *w*/*v*. In addition to UAE, an alternative extraction technique was used, ASE, with conventional organic solvents such as ethanol, acetone, and n-hexane mixtures, in order to compare the efficiency of conventional and greener solvents. ASE was performed at room temperature, approximately 1600 Psi, in three cycles for 15 min using a Thermo Scientific™ Dionex™ ASE™ 350 system (Thermo Scientific, Waltham, MA, USA).

### 3.7. Quantification of Lipophilic Compounds Through HPLC-DAD Analysis

The four compounds mentioned in the Introduction were selected as target compounds in the subsequent extraction experiments, and the total extraction yield of the four was used as an indicator to evaluate and optimize the extraction technology. The lycopene, β-carotene, lutein and tocopherol concentrations in each extract were determined according to the calibration curves of pure standards by plotting absorbance × concentration (µg/mL) using concentrations between 5 and 50 μg/mL. Linear regression equations (R^2^ > 0.999) were obtained for each calibration curve and used to determine the lycopene content (µg/mL) in each extract of the experimental procedure, along with total bioactive compound content (sum of all four compounds of interest). Results are expressed as micrograms of lycopene per gram dry weight (D.W.) and micrograms per gram for the total amount of the four targeted analytes. After scanning the two solvents for lycopene content, total bioactive compound content, and potential antioxidant activity, the best solvent combining high extraction efficiencies and antioxidant activity was chosen for optimization of UAE parameters. The analytical sample was prepared after proper dilution (1:2, *v*/*v*) with an acetone–methanol mixture and filtration through 0.22 µm PTFE filters (LLG Spheros, Meckenheim, Germany). A Shimadzu HPLC 20AD (Tokyo, Japan) system was equipped with an SPD-M20A diode array detector. Separation was carried out in isocratic mode on a Kromasil 100 C18 5 μm (250 × 4.6 mm i.d.) column with a mobile phase consisting of mixture of acetonitrile–methanol–dichloromethane (7:1:2, *v*/*v*/*v*) with 0.05% TEA and 0.1% BHT. The column temperature was maintained at 30 °C, flow rate was set to 0.8 mL/min, and the injection volume was 20 µL. The chromatograms were monitored in the wavelength range of 200–600 nm. Detection and identification of carotenoids was performed at the following wavelengths: lycopene (473 nm), β-carotene (458 nm), lutein (448 nm), and tocopherol (293 nm).

HPLC peaks were identified using carotenoid standards, and quantification was based on the calibration curves of the standards using the external standard method at the wavelength of maximum absorbance for each compound listed above.

### 3.8. Antioxidant Capacity

The measurement of the scavenging capacity of tomato residue extracts towards the ABTS cation radical (ABTS•+) was performed according to the method described by Vamanu et al., with some modifications [37]. ABTS cations were generated by the reaction between the stock solution of ABTS (7 mM in water) and 2.45 mM potassium persulfate. The stock solution thus obtained was kept at room temperature, in the dark, for at least 12 h for complete generation of free radicals. After that, the solution was diluted with methanol to an absorbance suitable for analytical determinations. The ABTS•+ solution (2.5 mL) was mixed with 0.1 mL extract solution and 0.4 mL of distilled water, and the decrease in absorbance was recorded after 6 min at a wavelength of 734 nm. The blank sample contained 0.1 mL of methanol instead of the sample. Antioxidant capacity is expressed in mM Trolox based on the trolox calibration curve: Y = 20431X − 0.0121, R^2^ = 0.997.

### 3.9. Statistical Analysis

All experiments were performed in triplicate, and the data were calculated using Microsoft Excel. All the results are presented as means ± standard deviation. Differences between variables were tested for significance using ANOVA and post hoc tests (Tukey’s HSD) and were considered significant at *p* < 0.05.

## 4. Conclusions

From the results obtained in the present study, it can be concluded that tomato waste can be an important source of liposoluble compounds with proven bioactive properties. The use of choline chloride–1,3-butanediol based NADESs as green solvents to support a more environmentally friendly alternative to many conventional solvents, which can be toxic and harmful to both humans and the environment, has demonstrated its unique ability to simultaneously extract carotenoids and tocopherols from dried powder tomato waste. The results led to obtaining an extract rich in carotenoids, such as lycopene, and tocopherol that possess potent antioxidant properties and protect cells from oxidative damage, support cardiovascular health, and improve skin health. Comparison with organic solvents confirmed that NADESs allowed extractions with comparable carotenoid content, but higher antioxidant capacity. Further studies on the biological activity of the obtained extracts and the stability of the extracted compounds in the NADESs are in progress. The final utility of these enriched NADES-based extracts will be in the development of biocompatible nanoformulations for pharmaceutical, cosmetics, and nanomedicine applications. Ongoing research is essential to fully exploit the potential of NADESs in nanotechnology and nanoformulations.

## Figures and Tables

**Table 1:** Concentration (μg/g DW) of lipophilic compounds and antioxidant capacity (expressed as mM Trolox eq.) in tomato waste extracts using UAE and NADESs: time of extraction 30 min, temperature 55 °C, ultrasound power level 100%, radiation frequency 37 Hz, S/L 1:15 (*w*/*v*).

Compounds	NADES 1	NADES 2
Lutein	2.16 ± 0.02	4.12 ± 0.08
β-carotene	141.64 ± 12.15	273.60 ± 1.32
Lycopene	147.67 ± 2.48	198.74 ± 10.28
Tocopherol	117.74 ± 3.95	nd
Total content	409.20 ± 18.76	476.57 ± 11.68
TEAC	65.11 ± 0.90	21.61 ± 0.78

nd—not detected.

**Table 2:** Concentration (μg/g DW) of lipophilic compounds and antioxidant capacity (expressed as mM Trolox eq.) in tomato waste extracts using different molar ratio for the components of NADES (HBA: HBD) with the following extraction parameters: time of extraction 30 min, temperature 55 °C, ultrasound power level 100%, radiation frequency 37 Hz, S/L 1:15 (*w*/*v*).

Compound/HBA:HBD Ratio *	2:3	1:2	1:3	1:4	1:5
lutein	1.36 ± 0.07	1.72 ± 0.03	1.3 ± 0.13	1.66 ± 0.12	1.72 ± 0.06
β-carotene	125.46 ± 5.19	118.45 ± 3.43	122.42 ± 6.58	144.15 ± 5.72	149.87 ± 1.14
lycopene	146.63 ± 8.23	127.96 ± 3.37	107.39 ± 10.64	126.32 ± 7.54	126.86 ± 4.38
tocopherol	98.86 ± 4.00	95.98 ± 4.44	104.44 ± 4.79	111.5 ± 5.39	103.24 ± 0.26
Total content	372.31 ± 17.49	344.11 ± 11.27	335.55 ± 22.14	383.63 ± 18.77	381.69 ± 5.84
TEAC	66.11 ± 0.71	61.61 ± 0.25	60.5 ± 0.88	68.2 ± 0.90	68.6 ± 0.52
F		*p*-value		F_crit_	
153.27		7.85 × 10^−10^		3.49	

* HBA:HBD ratio of 2:3; 1:2; 1:3; 1:4; 1:5.

**Table 3:** Concentration (μg/g DW) of lipophilic compounds and antioxidant capacity (expressed as mM Trolox eq.) in tomato waste extracts using different solid-to-liquid ratios maintaining the following parameters constant: time of extraction 30 min, temperature 55 °C ultrasound power level 100%, radiation frequency 37 Hz, HBA–HBD ratio 1:5.

Compound/S/L Ratio *	1:7.5	1:10	1:12.5	1:15	1:20
lutein	3.33 ± 0.19	2.53 ± 0.16	1.91 ± 0.05	1.60 ± 0.03	0.82 ± 0.07
β-carotene	143.61 ± 3.46	133.13 ± 4.33	137.69 ± 3.22	149.51 ± 1.14	171.12 ± 9.63
lycopene	155.66 ± 3.20	137.08 ± 1.89	140.31 ± 4.50	152.76 ± 2.20	176.19 ± 12.41
tocopherol	81.13 ± 4.37	79.56 ± 5.69	87.22 ± 2.36	101.76 ± 6.07	123.98 ± 7.09
Total content	383.73 ± 11.22	352.30 ± 12.07	367.13 ± 10.13	405.63 ± 9.44	472.11 ± 2.92
TEAC	67.01 ± 0.45	62.56 ± 0.22	63.05 ± 0.80	70.5 ± 0.44	73.5 ± 0.72
F		*p*-value		F_crit_	
79.18		3.56 × 10^−8^		3.49	

* S/L ratio of 1:7.5; 1:10; 1:12.5; 1:15; 1:20.

**Table 4:** Concentration (μg/g DW) of lipophilic compounds and antioxidant capacity (expressed as mM Trolox eq.) in tomato waste extracts obtained at different times of extraction (min): temperature 55 °C, ultrasound power level 100%, radiation frequency 37 Hz, HBA–ABD ratio 1:5, S/L 1:20 (*w*/*v*).

Compound/ Time of Extraction	T12	T24	T36	T48	T60
lutein	0.59 ± 0.14	0.62 ± 0.13	0.72 ± 0.03	0.65 ± 0.08	0.78 ± 0.18
β-carotene	163.65 ± 7.28	157.68 ± 7.10	162.34 ± 3.64	168.78 ± 2.20	160.47 ± 2.30
lycopene	181.38 ± 3.90	173.39 ± 9.07	175.01 ± 2.22	156.84 ± 5.66	157.54 ± 7.74
tocopherol	122.41 ± 5.69	128.02 ± 6.99	121.61 ± 7.06	130.02 ± 3.65	121.68 ± 5.67
Total content	468.03 ± 17.01	459.71 ± 23.29	459.68 ± 12.95	456.29 ± 11.59	440.47 ± 15.89
TEAC	77.06 ± 0.15	72.65 ± 0.81	73.05 ± 0.27	70.2 ± 0.11	63.3 ± 0.77
F		*p*-value		F_crit_	
694.6		1.03 × 10^−13^		3.49	

Time of extraction: T12 = 12 min, T24 = 24 min, T36 = 36 min, T48 = 48 min, T60 = 60 min.

**Table 5:** Concentration (μg/g DW) of lipophilic compounds and antioxidant capacity (expressed as mM Trolox eq.) in tomato waste extracts obtained at different extraction temperatures: time of extraction 12 min, ultrasound power level 100%, radiation frequency 37 Hz, HBA:ABD ratio 1:5, S/L 1:20 (*w*/*v*).

Compound/ Extraction Temperature	T25	T35	T45	T55	T65
lutein	0.39 ± 0.12	0.74 ± 0.02	0.97 ± 0.02	1.12 ± 0.02	0.86 ± 0.09
β-carotene	158.91 ± 7.84	167.80 ± 8.80	176.28 ± 2.08	199.33 ± 2.81	206.01 ± 2.31
lycopene	155.33 ± 9.59	163.82 ± 9.46	183.45 ± 1.16	201.15 ± 3.84	210.23 ± 2.98
tocopherol	115.59 ± 2.12	127.03 ± 6.46	126.04 ± 9.63	128.60 ± 6.43	133.36 ± 5.32
Total content	430.22 ± 19.67	459.39 ± 24.74	486.74 ± 12.89	530.20 ± 13.10	550.46 ± 10.70
TEAC	61.01 ± 0.18	70.56 ± 0.12	74.15 ± 0.91	78.5 ± 0.25	92.61 ± 0.19
F		*p*-value		F_crit_	
163.99		5.29 × 10^−10^		3.49	

Extraction temperature: T25 = 25 °C, T35 = 35 °C, T45 = 45 °C, T55 = 55 °C, T65 = 65 °C.

**Table 6:** Concentration (μg/g DW) of lipophilic compounds and antioxidant capacity (expressed as mM Trolox eq.) in tomato waste extracts obtained using different ultrasound power levels: time of extraction 12 min, temperature 65 °C, radiation frequency 37 Hz, HBA–ABD ratio 1:5, S/L 1:20 (*w*/*v*).

Compound US Power	T30	T50	T70	T90	T100
lutein	1.06 ± 0.11	1.20 ± 0.02	0.90 ± 0.06	0.88 ± 0.07	0.67 ± 0.17
β-carotene	179.13 ± 2.57	192.45 ± 1.17	206.95 ± 3.27	202.82 ± 1.57	192.50 ± 6.16
lycopene	187.37 ± 3.51	197.56 ± 0.35	215.13 ± 4.31	211.11 ± 0.62	202.54 ± 6.62
tocopherol	124.98 ± 6.79	124.68 ± 4.56	130.86 ± 8.97	131.76 ± 6.90	123.04 ± 4.01
Total content	492.54 ± 12.98	515.89 ± 6.10	553.84 ± 16.61	546.57 ± 9.16	518.75 ± 16.96
TEAC	74.99 ± 0.15	75.2 ± 0.72	93.84 ± 0.18	83.01 ± 0.45	76.18 ± 0.32
F		*p*-value		F_crit_	
1058.4		8.34 × 10^−15^		3.49	

Ultrasound power levels: T30 = 30%, T50 = 50%, T70 = 70%, T90 = 90%, T100 = 100%.

**Table 7:** ANOVA of experimental data.

Source of Variation	SS	df	MS	F	*p*-Value	F Crit
Rows	28,096.97	24	1170.707	5.064688	4.36 × 10^−8^	1.669456
Columns	448,706.4	3	149,568.8	647.0614	5.64 × 10^−52^	2.731807
Error	16,642.86	72	231.1509			
Total	493,446.3	99				

Rows represent the experimental conditions, columns represent the compounds.

**Table 8:** Concentration (μg/g DW) of lipophilic compounds in tomato waste extracts obtained using UAE and ASE methods. Extraction conditions are described in Section 3.

Solvents	NADES 1 ^a^	SFO ^a^	Organic Mixture ^b^	Ethanol 96% ^b^
lutein	0.90 ± 0.06	nd	9.27 ± 0.03	nd
β-carotene	206.95 ± 3.27	373.68 ± 4.83	380.48 ± 0.69	62.69 ± 7.23
lycopene	215.13 ± 4.31	486.76 ± 8.05	91.36 ± 0.33	8.19 ± 0.06
tocopherol	130.86 ± 8.97	2890.43 ± 10.98	84.32 ± 0.63	nd
Total content	553.84 ± 16.61	3750.87 ± 23.86	565.43 ± 1.68	70.88 ± 7.29

^a^ UAE, ^b^ ASE, SFO = sunflower oil, nd = not detected.

**Table 9:** Summary of studies on carotenoid recovery using innovative extraction technologies.

Method	Extraction Conditions	Extraction Yield mg/100 g Tomato Residue	Ref.
UAE	65 °C, 70 W, 12 min, S/L 1:20 *w*/*v*	20.69 ± 0.33 β-carotene 21.51 ± 0.43 lycopene 13.09 ± 0.90 tocopherol 0.09 ± 0.01 lutein	This study
PEF	1.0 kV/cm for 7.5 ms, 20–27 °C, S/L 1:10 (*w*/*v*)	14.31 ± 0.33 lycopene 17.77 ± 0.36 β-carotene	[29]
UAE	15 ± 5 °C, 90 W, 15–30 min, S/L 1:35 *w*/*v*	5.221–5.719 lycopene 0.442–0.490 β-carotene	[30]
SFE	86 °C, 34.47 MPa, 500 mL CO(2) flow rate 2.5 mL/min 3 g tomato residue	0.719 lycopene	[31]
SFE-CO_2_	300 bar, 80 °C, 130 g CO_2_/g residue	29.4 lycopene 6.97 β-carotene	[32]
Conventional extraction	Solvents: acetone–n-hexane (25:75), 40 °C, 40 min, S/L 3:50 (*w*/*v*)	300.85 lycopene 654.76 β-carotene	[33]
MAE	Solvent: NaCl aq. sol, 400 w, S/L 1:20 (*w*/*v*), 1600 W, (24 kJ equivalents), 15 s	13.592 lycopene	[34]

**Table 10:** Characteristics of NADESs used in this study.

NADESs	Component 1	Component 2	Density (g/cm^3^)	Molar Ratio
NADES-1	ChCl	1,3-Butanediol	1.0465	1:5
NADES-2	Oleic acid	Menthol	0.9061	1:1

ChCl—chlorine chloride.

## Data Availability

Data are contained within the article.
